# Effects of Dietary Glycine Supplementation on Growth Performance, Immunological, and Erythrocyte Antioxidant Parameters in Common Carp, *Cyprinus carpio*

**DOI:** 10.3390/ani13030412

**Published:** 2023-01-26

**Authors:** Marzieh Abbasi, Ali Taheri Mirghaed, Seyyed Morteza Hoseini, Hamid Rajabiesterabadi, Seyed Hossein Hoseinifar, Hien Van Doan

**Affiliations:** 1 Fisheries Department, Faculty of Natural Resources, University of Guilan, Sowmeh Sara 4361996196, Iran; 2 Department of Aquatic Animal Health, Faculty of Veterinary Medicine, University of Tehran, Tehran 14119963111, Iran; 3 Inland Waters Aquatics Resources Research Center, Iranian Fisheries Sciences Research Institute, Agricultural Research, Education and Extension Organization, Gorgan 4916687631, Iran; 4 Young Researchers and Elite Club, Azadshahr Branch, Islamic Azad University, Golestan 8998549617, Iran; 5 Department of Fisheries, Faculty of Fisheries and Environmental Sciences, Gorgan University of Agricultural Sciences and Natural Resources, Gorgan 4913815739, Iran; 6 Department of Animal and Aquatic Sciences, Faculty of Agriculture, Chiang Mai University, Chiang Mai 50200, Thailand

**Keywords:** amino acid, glutathione, nutrition, blood cells, skin mucus

## Abstract

**Simple Summary:**

Amino acids have various crucial roles in fish growth and health. Among them, the non-essential amino acids have been less studied in aquaculture, but they have many physiological roles. Glycine is a non-essential amino acid that is involved in glutathione structure, and is also involved in the antioxidant system in fish. Moreover, glycine stimulates the immune system. This study shows that a period of 8-week feeding with a diets supplemented with 5 g/kg glycine can improve growth performance, erythrocyte stability, and humoral and mucosal immunity in common carp. So, the present results can be used in carp diet production to support higher growth and health.

**Abstract:**

The effects of dietary glycine supplementation, 0 (control), 5 (5 GL), and 10 (10 GL) g/kg, have been investigated on growth performance, hematological parameters, erythrocyte antioxidant capacity, humoral and mucosal immunity in common carp, *Cyprinus carpio*. After eight weeks feeding, the 5 GL treatment exhibited significant improvement in growth performance and feed efficacy, compared to the control treatment. Red blood cell (RBC) and white blood cell (WBC) counts, hemoglobin, hematocrit, neutrophil and monocyte counts/percentages, RBC reduced glutathione (GSH) content, and skin mucosal alkaline phosphatase, peroxidase, protease, and lysozyme activities were similar in the glycine-treated fish and significantly higher than the control treatment. Blood lymphocyte percentage decreased in the glycine-treated fish, but lymphocyte count increased, compared to the control fish. RBC glutathione reductase activities in the glycine-treated fish were similar and significantly lower than the control treatment. The highest plasma lysozyme and alternative complement activities were observed in GL treatment. The glycine-treated fish, particularly 5 GL, exhibited significant improvement in RBC osmotic fragility resistance. Dietary glycine had no significant effects on RBC glutathione peroxidase activity, plasma immunoglobulin, eosinophil percentage/count, and hematological indices. In conclusion, most of the benefits of dietary glycine supplementation may be mediated by increased glutathione synthesis and antioxidant power.

## 1. Introduction

Dietary amino acids have important roles in fish growth and health. Fish depends on dietary supply to meet the requirement of certain amino acids, called essential amino acids. These amino acids cannot be synthetized in the fish body [1]. However, there are other amino acids that can be synthetized by fish, if certain substrates, enzymes, and cofactors are available. Most of the studies on amino acids’ requirement in fish have focused on the essential amino acids, but the non-essential amino acids should be focused as well. These amino acids, although can be synthetized in fish body, have many important physiological roles vital for fish health and welfare [2,3]. Thus, the conventional definition on essential/non-essential amino acids has been recently challenged by researchers.

Glycine is one of the non-essential amino acids in fish like other animals, but has many important physiological functions [2]. Glycine participates in collagen formation and 33% of collagen is made from this amino acid [4]. Moreover, glycine, in collaboration with glutamate and cysteine, participates in synthesizing of glutathione, a powerful and important antioxidant molecule [5]. Thus, glycine modulates the antioxidant system. This amino acid has various roles in immune function. It has been established that a drop in the blood serum glycine concentration increases serum resistance of various human pathogens [6]. A study on Nile tilapia, *Oreochromis niloticus*, has revealed that glycine metabolism pathways are of the most important pathways in prevention of mortality in the fish infected by *Edwardsiella tarda* [7].

There are a few studies regarding the effects of dietary glycine on fish, but the results are not consistent. For example, Nile tilapia has exhibited significant increase in growth performance, when fed a diet supplemented with 5 g/kg glycine. Dietary glycine has failed to affect various plasma and/or hepatic metabolites’ levels, antioxidant enzymes’ activities, lysozyme/myeloperoxidase activities, and malondialdehyde (MDA) content. Experimental challenge with *Streptococcus iniae* has shown that glycine failed to improve the disease resistance, and the other antioxidant/immunological responses were similar to pre-challenge, except an increase in plasma superoxide dismutase (SOD), glutathione reductase (GR), and myeloperoxidase activities [8]. A study on largemouth bass, *Micropterus salmoides*, has revealed that a plant-based diet supplemented with 20 g/kg glycine was beneficial to prevent growth retardation and intestinal villi shortage, compared to a fishmeal-based diet. However, no change in the hepatic SOD and glutathione peroxidase (GPx) activities and increases in hepatic MDA content and plasma alanine aminotransferase activity suggest possible hepatic problems in the fish [9]. Hoseini, Moghaddam [10] have shown that glycine supplementation has no significant effects on growth performance of beluga, *Huso huso*. White blood cell (WBC) count, plasma reduced glutathione (GSH) level, GPx, lysozyme, and complement activities increased at 2.5 and/or 5 g/kg glycine levels, implying the antioxidant and immunomodulation effects of glycine; however, significant increase in plasma MDA content at 5 and 10 g/kg glycine makes it hard to believe the antioxidant effects of glycine on this species. Our previous study on common carp, *Cyprinus carpio*, has shown that a 3-week period of feeding with 2.5–10 g/kg glycine is beneficial in improving the plasma lysozyme (5 and 10 g/kg glycine), GPx (2.5–10 g/kg glycine), glutathione-s-transferase (GST; 10 g/kg glycine) activities, and GSH (5 and 10 g/kg glycine) content, but decreases the plasma catalase (CAT; 5 and 10 g/kg glycine) activity and has no significant effects on the plasma MDA [11]. An 8-week feeding with the same diets have shown that glycine had no growth-promoting effects on the fish, although numerical improvements of ~10.5 and 12.5% were observed in the fish growth rate and feed efficiency, respectively. Plasma SOD, CAT, GPx, and GST remained unchanged, but plasma GSH and MDA increased and decreased, respectively [12]. These studies indicate that further evaluations are needed to improve the current knowledge regarding the effects of dietary glycine in fish. For example, the above-mentioned studies have focused on limited numbers of humoral innate immune parameters (lysozyme, complement, myeloperoxidase, respiratory burst activities, and WBC) and neglected others, such as skin mucosal immune parameters. This is an important topic considering several immunological roles of glycine reported in mammals [13] and its particular role in mucosal immunity observed in pigs [14]. Moreover, antioxidant effects of glycine have been only assessed in fish blood and liver, but it has been demonstrated that red blood cells (RBC), both in mammals and fish, actively pump glycine into their cytoplasm [15,16] to support glutathione synthesis and maintain internal antioxidant capacity. Glycine is also a key component of heme synthesis by participation in 5-aminolevulinic acid production; inhibition of glycine import by erythroids led to disrupted heme synthesis in mammals [17].

Considering limited information regarding the roles of dietary glycine in fish (compared to mammals), the present study was designed to assess the effects of dietary glycine on growth performance, the skin mucosal and humoral immune parameters, hematological parameters, RBC antioxidant capacity and propensity to hemolysis in common carp.

## 2. Materials and Methods

### 2.1. Diets

In light of the previous studies [11,12], we chose 5 (5 GL) and 10 (10 GL) g/kg glycine for supplementation, but reduced basal (endogenous) glycine level (from 19.8 to 16.1 g/kg) by reducing the amount of poultry by-product in the diet to find if glycine supplementation in plant-based diets (low poultry by-product) can support maximum growth. A control diet without glycine supplementation (CTL) was also included. Feedstuff (corn meal, wheat meal, soybean meal, cottonseed meal, fish canning by-product, and poultry by-product) were sieved (200 µ), mixed at the desired proportions (Table 1). Then, soybean oil, vitamin/mineral premixes, amino acids, cellulose, and glycine were added to the mixture. About 400 mL/kg water was added to the mixture and a dough was prepared, which was pelleted using a meat grinder. Dietary proximate composition was determined (three sample per diet) based on standard methods (kjeldahl method for protein; ether extraction for lipid; oven drying at 70 °C for moisture; furnace combustion for ash, and acid/base digestion for fiber) as described by Hoseini, Moghaddam [10]. Dietary amino acid profile was determined by an HPLC system (Waters Corporation, Milford, MA, USA). The samples were defatted with n-hexane and digested by HCl, followed by derivitization with phenyisothiocyanase, separation with PICO.TAG column, and detection in a dual λ absorbance detector [10].

### 2.2. Fish Rearing

Two hundred common carp juveniles were purchased from a local farm and stocked in one 1 m^3^ tank for 7 days, during which they were fed the CTL diet. After that, 90 healthy fish (24.5 ± 0.21 g) with a uniform size were selected and randomly distributed in 9 glass aquaria. The aquaria were filled with 50-L dechlorinated tap water, with continuous aeration. The stocking density was based on a previous study on the same fish species [12]. The fish were fed either of the above-mentioned diets, twice a day (2% of biomass), for 8 weeks. Every other week, the fish of each aquarium were bulk-caught and placed in a tank filled with water on a digital scale to record the tank biomass and the feed amounts adjustment. Water of the aquaria was renewed by 30% every day and the wastes were siphoned out. Water temperature, pH, dissolved oxygen, and total ammonia were 23.8 ± 0.85 °C, 7.74 ± 0.68, 6.78 ± 0.74 mg/L, and 1.53 ± 0.33 mg/L, respectively.

After 8 weeks rearing, weight gain (WG), specific growth rate (SGR), and feed conversion ratio (FCR) were recorded as follows:

WG (%) = 100 × [(final weight − initial weight)/initial weight](1)

FCR = feed intake/(final weight − initial weight)(2)

SGR (%/day) = 100 × [(Ln final weight − Ln initial weight)/rearing days](3)

### 2.3. Sample Collection and Processing

At the end of the rearing period, three fish were caught from each aquarium and anesthetized in 100 µL/L eugenol. Then, blood samples (1 mL per fish) were taken from the fish caudal vein, using heparinized syringes. A portion of 300 µL of the fresh blood samples was used for hematological examinations. Another portion of 200 µL of the fresh blood samples was used for osmotic fragility test of the RBC. To make the cell lysate, the packed RBC of the samples were frozen at −70 °C and added with two volumes of phosphate buffer (pH 7.0). After vortexing, the suspensions were centrifuged (6000× *g*; 7 min; 4 °C) and the supernatants were kept at −70 °C until analysis of the RBC GSH, GR, and GPx. To obtain plasma, 350 µL of the fresh blood samples were centrifuged (6000× *g*; 7 min; 4 °C) and the plasma samples were kept at −70 °C until analysis of lysozyme, alternative complement (ACH50), and total immunoglobulin (Ig).

The skin mucus samples were collected from three fish per tanks. The fish were anesthetized as mentioned above and the mucus samples were collected from the dorsolateral surface by scrubbing a spatula. The collected mucus samples were mixed with equal volume of Tris-buffered saline (TBS, 50 mM Tris–HCl, 150 mM NaCl, pH 8.0). After vortexing, the mixtures were centrifuged (13,000× *g*; 15 min; 4 °C) and the supernatants were kept at −70 °C until the mucosal immunological assays.

### 2.4. Analysis

#### 2.4.1. Hematological Examinations

RBC and WBC were counted using a Neubauer chamber, following dilution with the dacie solution. Hematocrit (Hct) and hemoglobin (Hb) were measured by centrifuging and cyanomethemoglobin methods, respectively. Mean corpuscular volume (MCV), mean corpuscular hemoglobin (MCH), and mean corpuscular hemoglobin concentration (MCHC) were calculated according to Dacie and Lewis [18]. Leukocyte differential count was performed by preparing blood slides stained by Giemsa.

#### 2.4.2. Plasma Immunological Parameters

Plasma lysozyme activity was determined based on a turbidimetric method, as described by Ellis [19]. *Micrococcus luteus* was used as the target and suspended in phosphate buffer (pH 6.2). To 1 mL of this suspension, 25 µL of the plasma sample was added and average decrease in optical density per minute was recorded over 5 min at 450 nm. Each 0.001 decrease in optical density per minute was considered as one unit of lysozyme activity.

Plasma ACH50 activity was measured based on a hemolytic method [20], using sheep RBC as the target in veronal buffer containing magnesium and gelatin (pH 7.0). Serially diluted plasma samples were added to the suspension of the RBC in the buffer and incubated at room temperature for 60 min. The plasma amount leading to 50% hemolysis was estimated and used to calculate ACH50 activity per mL plasma.

Plasma total Ig concentration was determined after precipitation with polyethylene glycol [21]. Equal amounts of the plasma and polyethylene glycol solution (12%) were mixed and kept under agitation at room temperature. After 2 h, the mixture was centrifuged (6000× *g*; 7 min; 4 °C) to precipitate Ig. Difference in the sample protein before and after the centrifugation was equal to the sample total Ig concentration.

#### 2.4.3. RBC Antioxidant Enzymes

The RBC antioxidant parameters were assessed according to Yousefi, Hoseini [22]. The RBC cell lysates were thawed and the solutions were centrifuged to precipitate any derbies. The supernatants were used for enzymatic assays. Commercial kits (Zellbio Co., GmbH, Deutschland, Germany) were used to assay the enzymes’ activity. GSH concentration was measured based on reaction with 5,5′-dithiobis-(2-nitrobenzoic acid) at 412 nm. The principal of GPx assay was based on adding GSH to the samples and conversion of GSH to oxidized glutathione (GSSG) by the sample GPx. The resultant GSSG is recycled to GSH by GR activity, which uses NADPH. The decrease in NADPH concentration is proportional to GPx activity and measurable at 340 nm. The same protocol is used for GR activity determination, but GSSG is added to the samples instead of GSH. The solution hemoglobin level was measured and the enzymes’ activities were expressed based on it.

#### 2.4.4. Osmotic Fragility Test

Osmotic fragility test of RBC was conducted according to Gao, Liu [23]. In brief, 25 µL of the packed fresh RBC was added to 1 mL of 0.1, 0.2, 0.3, 0.4, 0.5, 0.6, 0.7, 0.85% sodium chloride solution and left at room temperature for 90 min. Then, the suspensions were centrifuged (6000× *g*; 7 min; 4 °C) and the absorbance of the supernatants were measured at 492 nm. The optical densities were corrected based on the RBC count and MCH. The highest optical density was considered as 100% hemolysis and the other optical densities hemolysis rates were calculated based on it.

#### 2.4.5. Skin Mucus Immunological Parameters

Soluble protein concentrations of the skin mucus samples were determined based on Bradford [24]. The skin mucus peroxidase activity was determined according to Quade and Roth [25], using 3,3′,5,5′-tetramethylbenzidine hydrochloride and hydrogen peroxide as the substrates at 450 nm. The skin mucus protease activity was determined according to Zhang, Hu [26], using AZOcasein as the substrate at 350 nm. The skin mucus lysozyme activity was determined as described above. The skin mucus alkaline phosphatase (ALP) activity was determined using Parsazmun Co. kit (Tehran, Iran), according to Hoseinifar, Roosta [27].

### 2.5. Statistical Analysis

Before analysis, the averages of data per aquarium were calculated and used for statistical analysis (*n* = 3). After confirming normal distribution of the data by the Shapiro–Wilk test, and variance homogeneity by the Levene test, the data were subjected to one-way ANOVA. Significant differences among the treatments were determined by Tukey test. *p* < 0.05 was considered as significance and all analyses were performed in SPSS v.22.

## 3. Results

Growth performance of the fish is presented in Table 2. FCR in the 5 GL and 10 GL treatments were significantly lower than that of the CTL treatment. Final weight, WG, SGR in the 5 GL treatment were significantly higher than that of the CTL treatment. There was no mortality among the treatments.

RBC, Hct, and Hb of the 5 GL and 10 GL treatments were similar and significantly higher than those of the CTL treatment (Table 3). Blood MCV in the 10 GL treatment significantly decreased, compared to the CTL treatment. There were no significant differences in the blood MCH and MCHC among the treatments (Table 3).

Antioxidant parameters of RBC are shown in Table 3. GSH content significantly increased, as GR activity significantly decreased in the 5 GL and 10 GL treatments, compared to the CTL treatment. There was no significant difference in GPx activity among the treatments.

The results of osmotic fragility test are presented in Table 4. There were no significant differences in hemolysis rate among the treatments at NaCl concentrations of 0.1, 0.2, 0.7, and 0.85%. At NaCl concentration of 0.3–0.5%, hemolysis rate of the 5 GL treatment was significantly lower than the CTL treatment. The hemolysis rates of glycine-treated fish at NaCl concentration of 0.6% were significantly lower than those of the CTL treatment.

WBC and differential leukocyte counts are presented in Table 5. Dietary glycine supplementation significantly increased WBC count, and neutrophil and monocyte percentages/counts, compared to the CTL treatment. On the other hand, the glycine-treated fish had significantly lower lymphocyte percentages, but higher lymphocyte count, compared to the CTL fish. There were no significant differences in WBC and leukocyte differential counts between the 5 GL and 10 GL treatments. The blood eosinophil percentages/counts were similar among the treatments. Plasma immunological parameters are presented in Table 5. The glycine-treated fish exhibited significantly higher plasma lysozyme and ACH50 activities, compared to the CTL fish. The highest plasma lysozyme and ACH50 activities were observed in the 5 GL treatment. Dietary glycine supplementation had no significant effects on the plasma total Ig concentrations.

The skin mucosal immunological parameters of the fish are presented in Table 6. There were no significant differences in the mucosal soluble protein content, lysozyme, peroxidase, protease, and ALP activities between the 5 GL and 10 GL treatments; both exhibited significantly higher values, compared to the CTL treatment.

## 4. Discussion

Glycine contributes to synthesis of structural proteins and accounts for nearly 9% of the whole body protein in common carp [28]. In the present study, dietary glycine supplementation significantly improved growth performance and feed efficiency in common carp, which is not similar to our previous study on the same species [12]. The exact reasons for such a difference are not clear; it was expected that the lower glycine level in the control diet of the present study (1.61%), compared to the previous one (1.98%), made the difference. However, the growth performance of the control fish in the two studies was approximately similar. Thus, other factors might contribute to such a difference. One explanation may be change in the dietary amino acid compositions between the two studies. Lysine and methionine, two major growth-limiting amino acids, in the present study were higher than the previous one and sub-optimal dietary levels of these amino acids result in lower nitrogen, amino acid, and glycine retentions [29,30]. The present results are in line with the previous studies on Nile tilapia [8] and largemouth bass [9] that dietary glycine supplementation has improved the fish growth performance. Although non-essential amino acids can be synthetized in fish body, their synthesis rate may be insufficient to meet the biological requirements. Hence, the improved growth performance in glycine treatments in the present study can be due to higher glycine availability to meet growth and maintenance requirements.

Hematological studies give a sight regarding the fish health and gas mobilization status. Decrease in RBC count and hemoglobin content hinders tissue oxygenation and carbon dioxide clearance [31]. Therefore, a number of studies have assessed the role of nutritional manipulations [32,33,34], including amino acid supplementation [35,36,37], on hematological parameters in different fish species. Little is known regarding the role of glycine in hematological parameters in fish. A study on channel catfish, *Ictalurus punctatus*, has revealed that RBC actively transports glycine into the cytoplasm, which can be used for glutathione synthesis [15], as observed in mammals [17]. Due to the presence of hemoglobin, RBC are in constant threat of oxidation and glutathione has a crucial role in prevention of RBC oxidative stress [15]. According to the present results, the improvements in glutathione-related antioxidant parameters (increase in GSH content and decrease in GR activity) can be responsible for higher RBC count and resistance to hemolysis in the glycine-treated fish. It has been revealed that fish erythrocytes are very sensitive to oxidative conditions, under which a significant drop in GSH content (that has been due to oxidation) and increase in GPx, lipid peroxidation, and hemolysis occur [38,39,40]. No change in GPx activity in the present study suggests probably no difference in oxidative conditions among the treatment. Thus, higher GSH contents in 5 GL and 10 GL treatments can be due to improved glutathione synthesis under glycine availability. Increase in GSH synthesis can fulfil the cell biological requirement, so no need for further recycling of GSSG, which explains the decreases in GR activity in 5 GL and 10 GL treatments.

Leukocytes are important immune cells with diverse functions that help the host to combat foreign germs [41]. Increase in WBC after dietary supplementations has been found to augment disease resistance in different fish species [42,43]. Regarding glycine, dietary supplementation has been found to increase WBC in beluga [10], similar to the present results. Neutrophils are the first defending leukocytes, when fish encounter pathogen attacks. They kill pathogens by phagocytosis, which leads to the formation of reactive molecules [44]. Monocytes are another immune cells with phagocytic functions [45]. Due to the production of various pro-oxidant molecules by these cells, they need a strong antioxidant system to survive. Studies on human cells have revealed that glutathione is crucially necessary for viability and function of neutrophils and monocytes [46,47], but there are limited information regarding this topic in fish. Studies on carp have revealed that glutathione content increases in neutrophils during phagocytosis or lipopolysaccharide induction [48]. Exposure of Nile tilapia monocytes to a pro-oxidant agent (copper nanoparticle) has induced apoptosis, oxidative stress, and depletion of GSH content [45]. Accordingly, it is speculated that higher blood neutrophil and monocyte number/percentage in the glycine-treated fish in the present study was a consequence of improvement in the cells GSH content. However, glycine has no significant effects on blood lymphocyte population and the decrease in lymphocyte percentages in the glycine-treated fish was as a result of increase in the percentages of other leukocyte types. All together, these results suggest that dietary glycine supplementation improves the strength of the innate immunity system.

Lysozyme acts as a bactericidal agent that is secreted by blood neutrophils in the circulation [49]. Therefore, it is speculated that the increase in plasma lysozyme in the glycine-treated fish was due to the increase in the blood neutrophil population or their lysozyme production. Similarly, elevations in the plasma lysozyme activity have been accompanied by elevations in the blood neutrophil population, after adding various feed additives to fish diets [50,51]. Similar to the present study, dietary glycine supplementation has significantly increased plasma lysozyme activity in beluga [10] and common carp [11]. The complement system acts as a germ-killing and opsonizing agent in fish that consists of several proteins produced in the liver [52]. Dietary glycine supplementation have been found to increase the plasma ACH50 activity in beluga [10]; however, a short-term period of feeding with glycine-supplemented diets induced no significant changes in the plasma ACH50 activity in common carp [11]. The exact mechanism by which glycine improves the plasma ACH50 activity is not clear, but GSH may contribute, as dietary glutathione supplementation has significantly increased concentration of complement proteins in hemolymph of mitten crab, *Eriocheir sinensis*, which may be as a results of higher hepatopancreas health and protein synthesis caused by GSH availability [53]. Therefore, it is speculated that dietary glycine can increase GSH concentration, which in turn may be responsible for higher ACH50 activity in the present study. Moreover, properdin, a glycine-rich molecule that up-regulates the complement activity, has been identified in various tissues of fish such as the liver, skin, neutrophil, and monocyte [54,55]. This molecule has been identified in common carp [56], so it is speculated that glycine availability might support higher properdin production and increased ACH50 activity in the present study.

Skin mucosal immunity is very important to prevent diseases in aquaculture practice, as water serves as the main pathogen transmitter and a strong mucosal immunity can prevent the entrance of pathogens to new fish [57]. Skin mucosal lysozyme acts as a bactericidal agent, similar to plasma lysozyme [49]. Protease in the skin mucus kills pathogens, decreases mucus integrity, increases mucus layer sloughing, and activates other immune parameters [58]. Skin mucosal peroxidase participates in the formation of peroxidase–H_2_O_2_–halide complex, a strong bactericidal and cytotoxic agent [58]. ALP activity in the skin mucus can detoxify pro-inflammatory compounds created by microbes [59]. There are no similar studies for comparison, but the present results suggest that dietary glycine supplementation can improve the skin mucosal immunity in common carp.

## 5. Conclusions

In conclusion, dietary glycine supplementation can support GSH synthesis, which improves antioxidant capacity and hemolysis resistance in RBC. Moreover, dietary glycine supplementation increase the population of blood neutrophils and monocytes that can be due to protective role of GSH in these cells. Interestingly, glycine can improve several immune-related factors in the fish skin mucus that can be helpful in inhibition of pathogen entering fish body. Based on these benefits, dietary 5 g/kg glycine supplementation is recommended for common carp feed supplementation.

## Figures and Tables

**Table 1:** Compositions of the experimental diets containing grading levels of glycine.

Ingredients (g/kg)	CTL	5 GL	10 GL	Amino Acid Profile (%)	CTL	5 GL	10 GL
Corn meal	50	50	50	Glycine	1.61	2.07	2.74
Wheat meal	260	260	260	Arginine	2.47	2.35	2.56
Soybean meal ^1^	350	350	350	Serine	1.83	1.63	1.75
Soybean oil	63	63	63	Glutamic acid	8.52	8.06	8.08
Fish canning by-product ^2^	100	100	100	Histidine	0.87	0.92	0.80
Cotton seed meal ^3^	60	60	60	Isoleucine	1.62	1.57	1.64
Poultry by-product ^4^	80	80	80	Leucine	2.54	2.43	2.65
Vitamin premix ^5^	5	5	5	Lysine	2.45	2.40	2.39
Mineral premix ^6^	5	5	5	Methionine	1.10	1.19	1.11
Methionine ^7^	6	6	6	Cyctein	0.56	0.53	0.49
Lysine ^8^	6	6	6	Alanine	1.14	1.09	1.02
Cellulose ^9^	15	10	5	Phenylalanine	1.58	1.46	1.49
Glycine ^10^	0	5	10	Tyrosine	1.15	1.15	1.21
Proximate composition				Threonine	1.29	1.33	1.38
Moisture (g/kg)	106	102	107	Tryptophan	0.48	0.42	0.43
Crude protein (g/kg dry matter)	338 ± 1.62	343 ± 0.99	347 ± 2.32	Valine	1.71	1.63	1.67
Crude fat (g/kg dry matter)	129 ± 1.35	132 ± 0.92	131 ± 0.37				
Crude ash (g/kg dry matter)	57.9 ± 0.13	58.4 ± 0.10	58.1 ± 0.23				
Crude fiber (g/kg dry matter)	51.3 ± 1.46	44.5 ± 0.87	39.1 ± 2.13				

^1^ crude protein 42%; crude fat 1%; ^2^ crude protein 55%; crude fat 17%; ^3^ crude protein 39%; crude fat 2%; ^4^ crude protein 50%; crude fat 19%; ^5^ Amineh Gostar Co. (Tehran, Iran); the premix provided vitamins as follow (per kg diet): A: 1600 IU; D3: 500 IU; E: 20 mg; K: 24 mg; B3: 12 mg; B5: 40 mg; B2: 10 mg; B6: 5 mg; B1: 4 mg; H: 0.2 mg; B9: 2 mg; B12: 0.01 mg; C: 60 mg; Inositol: 50 mg; ^6^ Amineh Gostar Co. (Tehran, Iran); the premix provided minerals as follow (per kg diet): Se: 0.15 mg; Fe: 2.5 mg; Co: 0.04 mg; Mn: 5 mg; iodate: 0.05 mg; Cu: 0.5 mg; Zn: 6 mg; choline: 150 mg; ^7^ CheilJedang Co., Seul, Korea; ^8^ CheilJedang Co., Seul, Korea; ^9^ Sigma-Aldrich Co. (St. Louis, MO, USA); 99%; ^10^ Sigma-Aldrich Co. (St. Louis, MO, USA); 99%.

**Table 2:** Growth performance of common carp following eight weeks feeding with diets containing graded levels of glycine. Different letters within a row indicate significant differences among the treatments (mean ± SE; *n* = 3; Tukey).

	CTL	5 GL	10 GL	*p*-Value
Initial weight (g)	24.4 ± 0.59 a	24.6 ± 0.35 a	24.6 ± 0.23 a	0.921
Final weight (g)	48.8 ± 2.45 a	56.9 ± 1.78 b	54.8 ± 0.98 ab	0.049
FCR	1.49 ± 0.06 b	1.21 ± 0.05 a	1.26 ± 0.03 a	0.025
WG (%)	99.9 ± 5.22 a	131 ± 8.02 b	123 ± 4.31 ab	0.022
SGR (%/d)	1.24 ± 0.05 a	1.50 ± 0.06 b	1.43 ± 0.03 ab	0.018
Survival (%)	100	100	100	1.00

**Table 3:** Hematological and erythrocytes’ antioxidant parameters of common carp following eight weeks feeding with diets containing graded levels of glycine. Different letters indicate significant differences among the treatments (mean ± SE; *n* = 3; Tukey).

	CTL	5 GL	10 GL	*p*-Value
RBC (10^6^ cell/µL)	1.33 ± 0.01 a	1.52 ± 0.01 b	1.51 ± 0.01 b	<0.001
Hct (%)	33.3 ± 0.40 a	37.3 ± 0.08 b	36.1 ± 0.32 b	<0.001
Hb (g/dL)	7.79 ± 0.12 a	8.78 ± 0.15 b	8.46 ± 0.11 b	0.005
MCV (fL)	251 ± 1.82 b	245 ± 1.34 ab	241 ± 1.64 a	0.013
MCH (pg)	58.7 ± 1.14 a	57.7 ± 1.13 a	56.3 ± 1.00 a	0.365
MCHC (mg/dL)	23.4 ± 0.53 a	23.5 ± 0.36 a	23.4 ± 0.45 a	0.983
GSH (nM/mg Hb)	6.21 ± 0.02 a	7.88 ± 0.04 b	7.59 ± 0.08 b	<0.001
GR (U/mg Hb)	91.5 ± 0.29 b	75.8 ± 0.35 a	79.3 ± 0.41 a	<0.001
GPx (U/mg Hb)	79.2 ± 0.55 a	72.2 ± 0.46 a	74.2 ± 1.02 a	0.593

**Table 4:** Blood hemolysis rate of common carp following eight weeks feeding with diets containing graded levels of glycine. The hemolysis rates were calculated as percentages of the highest hemolysis among the samples. Different letters indicate significant differences among the treatments (mean ± SE; *n* = 3; Tukey).

NaCl concentration (%)	CTL	5 GL	10 GL	*p*-Value
0.1	93.2 ± 1.57 a	93.4 ± 2.20 a	91.5 ± 2.22 a	0.769
0.2	89.0 ± 1.80 a	86.8 ± 0.74 a	84.3 ± 1.83 a	0.189
0.3	81.1 ± 2.22 b	68.0 ± 2.83 a	78.6 ± 2.69 ab	0.026
0.4	72.3 ± 2.14 b	53.1 ± 2.03 a	65.6 ± 2.17 b	0.002
0.5	52.4 ± 1.45 b	37.0 ± 1.46 a	43.7 ± 3.34 ab	0.009
0.6	34.3 ± 0.22 b	18.0 ± 0.93 a	20.7 ± 0.73 a	<0.001
0.7	17.7 ± 1.29 a	15.4 ± 0.63 a	15.7 ± 0.20 a	0.247
0.85	13.9 ± 0.51 a	14.1 ± 1.01 a	15.0 ± 0.42 a	0.555

**Table 5:** Humoral immunological parameters of common carp following eight weeks feeding with diets containing graded levels of glycine. Different letters within a row indicate significant differences among the treatments (mean ± SE; *n* = 3; Tukey).

	CTL	5 GL	10 GL	*p*-Value
WBC (10^3^ cell/µL)	10.9 ± 0.08 a	14.3 ± 0.28 b	13.8 ± 0.24 b	<0.001
Lymphocyte (10^3^ cell/µL)	9.49 ± 0.11 a	10.4 ± 0.21 b	10.4 ± 0.18 b	0.013
Neutrophil (10^3^ cell/µL)	0.82 ± 0.04 a	2.65 ± 0.10 b	2.29 ± 0.11 b	<0.001
Monocyte (10^3^ cell/µL)	0.42 ± 0.03 a	0.95 ± 0.08 b	0.92 ± 0.03 b	0.001
Eosinophil (10^3^ cell/µL)	0.16 ± 0.01 a	0.21 ± 0.01 a	0.22 ± 0.04 a	0.327
Lymphocyte (%)	87.3 ± 0.51 b	73.4 ± 0.56 a	75.2 ± 0.73 a	<0.001
Neutrophil (%)	7.44 ± 0.48 a	18.4 ± 0.40 b	16.6 ± 0.62 b	<0.001
Monocyte (%)	3.78 ± 0.29 a	6.67 ± 0.58 b	6.67 ± 0.33 b	0.004
Eosinophil (%)	1.44 ± 0.11 a	1.44 ± 0.11 a	1.56 ± 0.29 a	0.897
Lysozyme (U/mL)	26.3 ± 0.47 a	50.4 ± 0.84 c	34.8 ± 0.29 b	<0.001
ACH50 (U/mL)	181 ± 4.56 a	397 ± 4.57 c	283 ± 2.47 b	<0.001
Total Ig (g/L)	7.87 ± 0.53 a	7.52 ± 0.65 a	8.17 ± 0.73 a	0.786

**Table 6:** The skin mucosal immunological parameters of common carp following eight weeks feeding with diets containing graded levels of glycine. Different letters indicate significant differences among the treatments (mean ± SE; *n* = 3; Tukey).

	CTL	5 GL	10 GL	*p*-Value
Soluble protein (mg/dL)	54.3 ± 1.26 a	77.1 ± 2.51 b	72.0 ± 2.03 b	<0.001
Proxidase (U/mg Pr)	3.96 ± 0.15 a	5.40 ± 0.13 b	5.66 ± 0.09 b	<0.001
Protease (U/mg Pr)	40.6 ± 0.29 a	63.6 ± 0.87 b	62.0 ± 0.19 b	<0.001
ALP (U/mg Pr)	120 ± 3.35 a	155 ± 3.09 b	155 ± 4.30 b	0.001
Lysozyme (U/mg Pr)	29.1 ± 0.53 a	38.7 ± 0.91 b	40.5 ± 1.16 b	<0.001

## Data Availability

The data of this study is available upon request from the corresponding author.

## References

[1] Mai K., Xue M., He G., Xie S.Q., Kaushik S.J., Hardy R.W., Kaushik S.J. (2022). Chapter 4—Protein and amino acids. Fish Nutrition.

[2] Li P., Mai K.S., Trushenski J., Wu G.Y. (2009). New developments in fish amino acid nutrition: Towards functional and environmentally oriented aquafeeds. Amino Acids..

[3] Hoseini S.M., Reverter M., Gupta S.K., Giri S.S. (2021). Health-promoting effects of amino acids in fish. Biotechnological Advances in Aquaculture Health Management.

[4] Li P., Wu G. (2018). Roles of dietary glycine, proline, and hydroxyproline in collagen synthesis and animal growth. Amino Acids..

[5] Pérez-Torres I., María Zuniga-Munoz A., Guarner-Lans V. (2017). Beneficial effects of the amino acid glycine. Mini. Rev. Med. Chem..

[6] Kou T.-S., Wu J.-H., Chen X.-W., Chen Z.-G., Zheng J., Peng B. (2022). Exogenous glycine promotes oxidation of glutathione and restores sensitivity of bacterial pathogens to serum-induced cell death. Redox Biol..

[7] Yang D.-X., Yang M.-J., Yin Y., Kou T.-S., Peng L.-T., Chen Z.-G. (2021). Serine metabolism tunes immune responses to promote *Oreochromis niloticus* survival upon *Edwardsiella tarda* infection. mSystems..

[8] Xie S., Zhou W., Tian L., Niu J., Liu Y. (2016). Effect of N-acetyl cysteine and glycine supplementation on growth performance, glutathione synthesis, anti-oxidative and immune ability of Nile tilapia, *Oreochromis niloticus*. Fish Shellfish. Immunol..

[9] Rossi W., Allen K.M., Habte-Tsion H.-M., Meesala K.-M. (2021). Supplementation of glycine, prebiotic, and nucleotides in soybean meal-based diets for largemouth bass (*Micropterus salmoides*): Effects on production performance, whole-body nutrient composition and retention, and intestinal histopathology. Aquaculture.

[10] Hoseini S.M., Moghaddam A.A., Ghelichpour M., Pagheh E., Haghpanah A., Gharavi B. (2022). Dietary glycine supplementation modulates antioxidant and immune responses of beluga, *Huso huso*, juveniles. Aquac. Rep..

[11] Hoseini S.M., Paolucci M., Arghideh M., Hosseinpour Delavar F., Zavvar F., Hoseinifar S.H. (2022). Effects of dietary glycine administration on biochemical responses to ammonia toxicity in common carp, *Cyprinus carpio*. Aquac. Res..

[12] Hoseini S.M., Majidiyan N., Mirghaed A.T., Hoseinifar S.H., Van Doan H. (2022). Dietary glycine supplementation alleviates transportation-induced stress in common carp, *Cyprinus carpio*. Aquaculture..

[13] Li P., Yin Y.-L., Li D., Woo Kim S., Wu G. (2007). Amino acids and immune function. Br. J. Nutr..

[14] Ji Y., Fan X., Zhang Y., Li J., Dai Z., Wu Z. (2021). Glycine regulates mucosal immunity and the intestinal microbial composition in weaned piglets. Amino Acids..

[15] Angermeier S.M., Shepard M.D., Tunnicliff G. (1996). Glycine transport by the red cells of channel catfish. Can. J. Zool..

[16] Kim K.M., Kingsmore S.F., Han H., Yang-Feng T.L., Godinot N., Seldin M.F. (1994). Cloning of the human glycine transporter type 1: Molecular and pharmacological characterization of novel isoform variants and chromosomal localization of the gene in the human and mouse genomes. Mol. Pharmacol..

[17] Winter M., Funk J., Körner A., Alberati D., Christen F., Schmitt G. (2016). Effects of GlyT1 inhibition on erythropoiesis and iron homeostasis in rats. Exp. Hematol..

[18] Dacie J., Lewis S. (1996). Practical Hematology.

[19] Ellis A.E., Stolen J.S. (1990). Lysozyme assays. Techniques in Fish Immunology.

[20] Yano T., Stolen J.S. (1992). Assays of hemolytic complement activity. Techniques in Fish Immunology.

[21] Siwicki A., Anderson D., Siwicki A., Anderson D., Waluga J. (1993). Nonspecific defense mechanisms assay in fish: II. Potential killing activity of neutrophils and macrophages, lysozyme activity in serum and organs and total immunoglobulin level in serum. Fish Disease Diagnosis and Prevention Methods.

[22] Yousefi M., Hoseini S.M., Vatnikov Y.A., Nikishov A.A., Kulikov E.V. (2018). Thymol as a new anesthetic in common carp (*Cyprinus carpio*): Efficacy and physiological effects in comparison with eugenol. Aquaculture.

[23] Gao Y.-J., Liu Y.-J., Chen X.-Q., Yang H.-J., Li X.-F., Tian L.-X. (2016). Effects of graded levels of histidine on growth performance, digested enzymes activities, erythrocyte osmotic fragility and hypoxia-tolerance of juvenile grass carp *Ctenopharyngodon idella*. Aquaculture.

[24] Bradford M.M. (1976). A rapid and sensitive method for the quantitation of microgram quantities of protein utilizing the principle of protein-dye binding. Anal Biochem..

[25] Quade M.J., Roth J.A. (1997). A rapid, direct assay to measure degranulation of bovine neutrophil primary granules. Vet Immunol. Immunopathol..

[26] Zhang W.-W., Hu Y.-H., Wang H.-L., Sun L. (2009). Identification and characterization of a virulence-associated protease from a pathogenic *Pseudomonas fluorescens* strain. Vet Microbiol..

[27] Hoseinifar S.H., Roosta Z., Hajimoradloo A., Vakili F. (2015). The effects of Lactobacillus acidophilus as feed supplement on skin mucosal immune parameters, intestinal microbiota, stress resistance and growth performance of black swordtail (*Xiphophorus helleri*). Fish Shellfish Immunol..

[28] Fu C., Cui Y., Hung S.S.O., Zhu Z. (2000). Whole-body amino acid pattern of F4 human growth hormone gene-transgenic red common carp (*Cyprinus carpio*) fed diets with different protein levels. Aquaculture.

[29] Gan L., Liu Y.J., Tian L.X., Yue Y.R., Yang H.J., Liu F.J. (2013). Effects of dissolved oxygen and dietary lysine levels on growth performance, feed conversion ratio and body composition of grass carp, *Ctenopharyngodon idella*. Aquacult. Nutr..

[30] Yun Y., Song D., He Z., Mi J., Wang L., Nie G. (2022). Effects of methionine supplementation in plant protein based diet on growth performance and fillet quality of juveniles Yellow River carp (*Cyprinus carpio haematopterus*). Aquaculture.

[31] Fazio F. (2019). Fish hematology analysis as an important tool of aquaculture: A review. Aquaculture.

[32] Mohseni M., Hamidoghli A., Bai S.C. (2021). Organic and inorganic dietary zinc in beluga sturgeon (*Huso huso*): Effects on growth, hematology, tissue concertation and oxidative capacity. Aquaculture.

[33] Mohseni M., Saltanat N.L., Rastravan M.E., Golalipour Y. (2021). Effects of betaine supplementation in plant-protein-based diets on growth performance, haemato-immunological parameters, antioxidant status and digestive enzyme activities of juvenile Caspian trout (*Salmo trutta*, Kessler, 1877). Aquacult. Nutr..

[34] Aftabgard M., Salarzadeh A., Mohseni M., Bahri Shabanipour A.H., Zorriehzahra M.E.J. (2019). The combined efficiency of dietary isomaltooligosaccharides and *Bacillus* spp. on the growth, hemato-serological, and intestinal microbiota indices of Caspian brown trout (*Salmo trutta caspius* Kessler, 1877). Probiotics Antimicrob. Proteins.

[35] Michelato M., Zaminhan M., Boscolo W.R., Nogaroto V., Vicari M., Artoni R.F. (2017). Dietary histidine requirement of Nile tilapia juveniles based on growth performance, expression of muscle-growth-related genes and haematological responses. Aquaculture.

[36] Machado M., Azeredo R., Fontinha F., Fernández-Boo S., Conceição L.E.C., Dias J. (2018). Dietary methionine improves the European seabass (*Dicentrarchus labrax*) immune status, inflammatory response, and disease resistance. Front. Immunol..

[37] Sharf Y., Khan M.A. (2021). Requirement of fingerling *Channa punctatus* (Bloch) for dietary lysine based on growth, feed conversion and lysine retention efficiency, RNA/DNA ratio, haematological parameters and serum antioxidant activity. Aquacult. Nutr..

[38] Li H.-T., Feng L., Jiang W.-D., Liu Y., Jiang J., Li S.-H. (2013). Oxidative stress parameters and anti-apoptotic response to hydroxyl radicals in fish erythrocytes: Protective effects of glutamine, alanine, citrulline and proline. Aquat. Toxicol..

[39] Gwoździński K., Roche H., Pérès G. (1992). The comparison of the effects of heavy metal ions on the antioxidant enzyme activities in human and fish *Dicentrarchus labrax* erythrocytes. Comp. Biochem. Physiol. Part C Comp. Pharmacol..

[40] Janssens B.J., Le Gall R., Rees J.F. (2002). Peroxide-triggered erythrocytes haemolysis as a model for the study of oxidative damage in marine fishes. J. Fish Biol..

[41] Maisey K., Imarai M. (2011). Diversity of teleost leukocyte molecules: Role of alternative splicing. Fish Shellfish Immunol..

[42] Koch J.F.A., de Oliveira C.A.F., Zanuzzo F.S. (2021). Dietary β-glucan (MacroGard®) improves innate immune responses and disease resistance in Nile tilapia regardless of the administration period. Fish Shellfish Immunol..

[43] Yousefi M., Ghafarifarsani H., Hoseinifar S.H., Rashidian G., Van Doan H. (2021). Effects of dietary marjoram, *Origanum majorana* extract on growth performance, hematological, antioxidant, humoral and mucosal immune responses, and resistance of common carp, *Cyprinus carpio* against *Aeromonas hydrophila*. Fish Shellfish Immunol..

[44] Havixbeck J.J., Rieger A.M., Wong M.E., Hodgkinson J.W., Barreda D.R. (2016). Neutrophil contributions to the induction and regulation of the acute inflammatory response in teleost fish. J. Leukoc. Biol..

[45] Chen J., Dong Z., Lei Y., Yang Y., Guo Z., Ye J. (2022). β-glucan mitigation on toxicological effects in monocytes/macrophages of Nile tilapia (*Oreochromis niloticus*) following copper exposure. Fish Shellfish Immunol..

[46] Seres T., Knickelbein R.G., Warshaw J.B., Johnston R.B. (2000). The phagocytosis-associated respiratory burst in human monocytes is associated with increased uptake of glutathione1. J. Immunol..

[47] Carr A.C., Winterbourn C.C. (1997). Oxidation of neutrophil glutathione and protein thiols by myeloperoxidase-derived hypochlorous acid. Biochem, J..

[48] Saeij J.P.J., van Muiswinkel W.B., van de Meent M., Amaral C., Wiegertjes G.F. (2003). Different capacities of carp leukocytes to encounter nitric oxide-mediated stress: A role for the intracellular reduced glutathione pool. Dev. Comp. Immunol..

[49] Li L., Cardoso J.C.R., Félix R.C., Mateus A.P., Canário A.V.M., Power D.M. (2021). Fish lysozyme gene family evolution and divergent function in early development. Dev. Comp. Immunol..

[50] Dadras H., Hayatbakhsh M.R., Shelton W.L., Golpour A. (2016). Effects of dietary administration of Rose hip and Safflower on growth performance, haematological, biochemical parameters and innate immune response of Beluga, *Huso huso* (Linnaeus, 1758). Fish Shellfish Immunol..

[51] Estaiano de Rezende R.A., Soares M.P., Sampaio F.G., Cardoso I.L., Ishikawa M.M., Lima Dallago B.S. (2021). Phytobiotics blend as a dietary supplement for Nile tilapia health improvement. Fish Shellfish Immunol..

[52] Nakao M., Tsujikura M., Ichiki S., Vo T.K., Somamoto T. (2011). The complement system in teleost fish: Progress of post-homolog-hunting researches. Dev. Comp. Immunol..

[53] Liu J.-D., Chi C., Zheng X.-C., Xu C.-Y., Zhang C.-Y., Ye M.-W. (2019). Effect of dietary glutathione supplementation on the immune responses and the fatty acid and amino acid composition in Chinese mitten crab, *Eriocheir sinensis*. Aquac. Rep..

[54] Gonzalez S.F., Chatziandreou N., Nielsen M.E., Li W., Rogers J., Taylor R. (2007). Cutaneous immune responses in the common carp detected using transcript analysis. Mol. Immunol..

[55] Zhang Y., Chen J., Yao F., Ji D., Li H., Zhang S. (2013). Expression and functional analysis of properdin in zebrafish *Danio rerio*. Dev. Comp. Immunol..

[56] Yoshioka K., Kato-Unoki Y., Nagasawa T., Somamoto T., Nakao M. (2016). Carp properdin: Structural and functional diversity of two isotypes. Immunobiology.

[57] Noga E.J. (2010). Fish Disease: Diagnosis and Treatment.

[58] Guardiola F.A., Cuesta A., Arizcun M., Meseguer J., Esteban M.A. (2014). Comparative skin mucus and serum humoral defence mechanisms in the teleost gilthead seabream (*Sparus aurata*). Fish Shellfish Immunol..

[59] Lallès J.-P. (2019). Biology, environmental and nutritional modulation of skin mucus alkaline phosphatase in fish: A review. Fish Shellfish Immunol..

